# Accessible Spinal Surgery: Transformation Through the Implementation of Exoscopes As Substitutes for Conventional Microsurgery in Low- and Middle-Income Settings

**DOI:** 10.7759/cureus.45350

**Published:** 2023-09-16

**Authors:** Emir Begagić, Hakija Bečulić, Rasim Skomorac, Mirza Pojskić

**Affiliations:** 1 Department of General Medicine, School of Medicine, University of Zenica, Zenica, BIH; 2 Department of Neurosurgery, Cantonal Hospital Zenica, Zenica, BIH; 3 Department of Neurosurgery, University Hospital Marburg, Marburg, DEU

**Keywords:** exoscope, humanitarian health, new technologies in neurosurgery, low- and middle-income country, spinal cord tumor surgery

## Abstract

Advancements in neurosurgical visualization have been made possible by the introduction of the operating microscope (OM) and the emergence of exoscopic technology (EX). Both OMs and EXs provide enhanced magnification and illumination, but they come with their own set of advantages and disadvantages. OMs provide high-quality magnification and illumination and have been used successfully in a variety of surgical procedures. They can be customized to fit the specific needs of the surgeon and are a well-established technology. However, they can be bulky, expensive, and cause discomfort during extended procedures. EXs provide high-definition magnification and illumination, improved depth perception and ergonomics, and can be cost-effective. They can be customized to fit the specific needs of the surgeon and can be made using locally available materials, reducing the need for expensive imports. However, they may require adjustment and have a learning curve for surgeons who are used to operating with OMs. Additionally, they may have limited availability in some healthcare settings. The choice between OMs and EXs will depend on the specific needs of the surgeon and the healthcare setting. The integration of 3D EX systems has revolutionized neurosurgery, offering improved depth perception and ergonomics. EX's cost-effectiveness addresses accessibility concerns, making it an attractive alternative, particularly for low and middle-income healthcare settings. The exoscope seems to be a safe alternative compared to an operative microscope for the most common brain and spinal procedures. The exoscope may help expand access to neurosurgical care and training worldwide. In conclusion, both technologies have their own set of advantages and disadvantages, and the choice between them will depend on the specific needs of the surgeon and the healthcare setting.

## Editorial

Advancements in visual technology have significantly impacted the field of neurosurgery, particularly with the introduction of the operating microscope (OM) in the late 1950s. This innovation gained momentum in microsurgery by 1962 and extended to spinal surgery in 1977, offering high-quality magnification, illumination, and depth perception [1]. However, these advantages come with inherent drawbacks. OMs have limited mobility due to their fixed positioning, which can hinder the surgeon's flexibility and range of movement during intricate procedures. The substantial costs associated with procuring and maintaining OMs restrict their accessibility, often limiting their use to well-funded healthcare institutions. Prolonged procedures conducted under an OM can lead to surgeon discomfort and physical strain, potentially affecting procedural outcomes. Additionally, OMs may have limitations in providing a comprehensive view for the entire surgical team, limiting their potential for educational purposes [2].

In response to these challenges and the quest for enhanced surgical visualization, a novel solution emerged in the form of exoscopic technology (EX). This revolutionary system involves positioning a high-definition scope externally, projecting an enlarged view of the surgical field onto a 2D (two-dimensional) or 3D (three-dimensional) high-resolution monitor. Recent advancements have led to the development of 3D EX systems, which significantly enhance depth perception for surgeons [3]. The integration of 3D EX systems into neurosurgical practice, particularly in cranial and spinal surgeries, presents a promising avenue that could potentially rival the established utility of the conventional OM. Surgeons' experiences with the 3D EX have yielded favorable results, indicating improved ergonomics, reduced intraoperative fatigue, and enhanced surgical posture. Furthermore, the introduction of 3D visualization offers the advantage of providing a comprehensive and detailed view of the surgical field, benefiting all members of the operating room team and serving as an invaluable educational tool [3].

As ongoing research delves into the capabilities and outcomes of 3D EX technology across various neurosurgical procedures (i.e., posterior lumbar discectomy, anterior cervical discectomy with fusion, spinal tumors), it becomes increasingly crucial to critically evaluate its effectiveness and safety compared to the traditional OM. This evaluation is paramount in paving the way for the potential integration of the 3D EX as a viable alternative in the field of spinal surgery. The utilization of EXs in the realm of neurosurgery introduces a range of models, each carrying distinct advantages. Particularly notable are models like ViTOM® and ORBEYE®, which offer features such as robotic arms and advanced optics. Diverse modes, including HD 3D, HD 2D, and 3D 4K, provide neurosurgeons with adaptable visualization options catering to specific needs. Comparative assessments of video quality between EXs and traditional OMs yield varying results, with certain studies favoring EXs for superior visual quality and enhanced surgeon comfort. One area where EXs excel is in ergonomic design, contributing to reduced surgeon fatigue and optimized efficiency [4,5]. The availability of advanced surgical equipment is often limited in low- and middle-income settings. Magnification loupes could be a viable solution due to their affordability and portability. However, ergonomic feasibility and better magnification potential are offered by EXs. Hand-made EXs can provide similar benefits as standard EXs, such as high-quality magnification and illumination, but at a lower cost. These cost-effective options can be tailored to meet the specific needs of the surgeon and can be created using locally available materials, thus reducing the need for expensive imports. Moreover, they can be serviced and maintained locally, minimizing the need for expensive repairs and replacements. Therefore, in low- and middle-income settings, creating hand-made EXs and modifying existing resources could be a better alternative to magnification loupes.

Furthermore, EXs contribute significantly to the educational aspect of neurosurgery by offering detailed anatomical visualization [4]. This is particularly advantageous in procedures involving nerve and vascular-related tumor resections. While instances of switching back to traditional OMs during surgery have been noted, EXs exhibit the potential in streamlining operative workflows. However, opinions on efficiency and team involvement exhibit variability across studies, reflecting the ongoing exploration of EX benefits within the realm of neurosurgery. EXs have emerged as a promising alternative to traditional OMs, addressing some of these limitations. EX technology allows for external visualization through high-definition cameras that project images onto monitors, liberating surgeons from the constraints of fixed positions and offering improved mobility [3,4]. Enhanced mobility can potentially reduce fatigue and enhance ergonomics for surgeons, contributing to better procedural outcomes [1]. EX systems are often more cost-effective than OMs, which could democratize advanced surgical techniques and extend their benefits to a wider range of healthcare settings, including those in low- and middle-income countries. However, the adoption of EX technology introduces challenges such as video quality, as the external projection may not match the clarity of field offered by OMs. Additionally, surgeons may need to navigate a learning curve when transitioning from OMs to EX, requiring training to ensure optimal utilization of this innovative technology [4].

Future research in the domain of EX neurosurgery holds the potential to significantly shape the trajectory of surgical innovation. Comparative studies are essential to comprehensively assess the extended outcomes of EX procedures, shedding light on factors such as long-term patient recovery and recurrence rates. Expanding the scope of investigation beyond spinal surgeries to encompass a diverse array of neurosurgical procedures is crucial to fully understand the versatility and efficacy of EX techniques. Additionally, an emphasis on patient-centric parameters, such as the duration of the surgical procedure, intraoperative blood loss, incidence of complications, management of postoperative pain, overall quality of life, and levels of patient satisfaction, will yield valuable insights into the comprehensive ramifications of EX interventions [3-5]. Investigating the learning curve associated with EX procedures and establishing standardized training protocols are imperative steps toward ensuring skill acquisition and proficiency among surgeons. A comprehensive cost-benefit analysis is warranted to evaluate the economic implications of adopting EX technology, encompassing equipment costs, training expenditures, and potential improvements in patient outcomes. The global accessibility of EX technology, particularly in low- and middle-income countries, merits dedicated research efforts to identify strategies for bridging the accessibility gap and democratizing advanced surgical techniques. Collaborative initiatives involving neurosurgeons, engineers, and technologists should be promoted to continually refine EX design, incorporating innovative features that cater to the evolving needs of the neurosurgical community. Exploring the firsthand experiences and preferences of surgeons who have embraced EX techniques will provide invaluable insights into the practical implications, benefits, and challenges associated with their adoption. Investigating the impact of EX technology on education and awareness is essential for fostering informed decision-making and facilitating patient engagement in their care journeys. Lastly, the integration of artificial intelligence and machine learning with EX technology holds immense promise in enhancing real-time surgical assistance, anatomical recognition, and procedural guidance [5].

In conclusion, the evolution of EX techniques represents a significant stride in the realm of spinal surgery, offering a potential solution to the limitations posed by traditional operating microscopes. The integration of 3D EX systems enhances surgical visualization and holds promise for various neurosurgical procedures, including spinal surgeries. Comparative assessments highlight the favorable outcomes of EX in terms of ergonomics, surgical efficiency, and educational benefits. Moreover, the cost-effectiveness and adaptability of EX address accessibility challenges, particularly in low- and middle-income settings. As ongoing research advances our understanding of EX technology, the feasibility of EX spinal surgery emerges as a necessity in resource-constrained environments. This transformation not only improves surgical techniques but also paves the way for a more inclusive and accessible future in spinal surgery.
